# Progress and challenges in the development of advanced pancreatic cancer organoids

**DOI:** 10.1186/s13046-026-03717-3

**Published:** 2026-05-01

**Authors:** Katja Detert, Alban Piotrowsky, Luigi Marongiu, Christian Leischner, Ulrich M. Lauer, Sascha Venturelli, Markus Burkard

**Affiliations:** 1 Department of Nutritional Biochemistry, University of Hohenheim, Garbenstrasse 30, Stuttgart, 70599 Germany; 2 Department of Medical Oncology and Pneumology, Virotherapy Center Tübingen (VCT), Medical University Hospital, Otfried-Mueller-Strasse 27, Tuebingen, 72076 Germany; 3 German Cancer Consortium (DKTK), German Cancer Research Center (DKFZ), Partner Site Tuebingen, Tuebingen, 72070 Germany; 4 Department of Vegetative and Clinical Physiology, Institute of Physiology, University of Tuebingen, Wilhelmstrasse 56, Tuebingen, 72074 Germany

**Keywords:** Organoids, Pancreatic cancer, Hypoxia, Pancreatic ductal adenocarcinoma, Hypoxia-inducible factor, Extracellular matrices

## Abstract

Pancreatic ductal adenocarcinoma (PDAC) is an aggressive malignancy with poor prognosis and rising incidence. Late detection and limited responsiveness to standard treatment translates into a 5-year overall survival of less than 12%. The pathology contributes to a desmoplastic tumor microenvironment that creates a physical barrier, leading to a dense, hypoxic environment that promotes further tumorigenesis, limited immunogenicity, and chemoresistance, resulting in a still significant translational gap in PDAC research. Feasible techniques to further elucidate tumorigenesis are indispensable because of the frequently limited predictive value of current preclinical models. PDAC organoids offer a powerful tool that can be rapidly generated from resected tumors and biopsies. This review summarizes the current technical and scientific knowledge and highlights the importance of the tumor microenvironment, the use of realistic oxygen conditions, and the role of the hypoxia-inducible factors. Additionally, various protocols based on different media and scaffolds are displayed, and it is illustrated how PDAC organoids can help to improve both diagnosis and treatment options. Finally, critical bottlenecks in modeling PDAC tumor-stromal interactions are identified, and integrated co-culture platforms are proposed as a promising solution for translational applications.

**Graphical Abstract:**

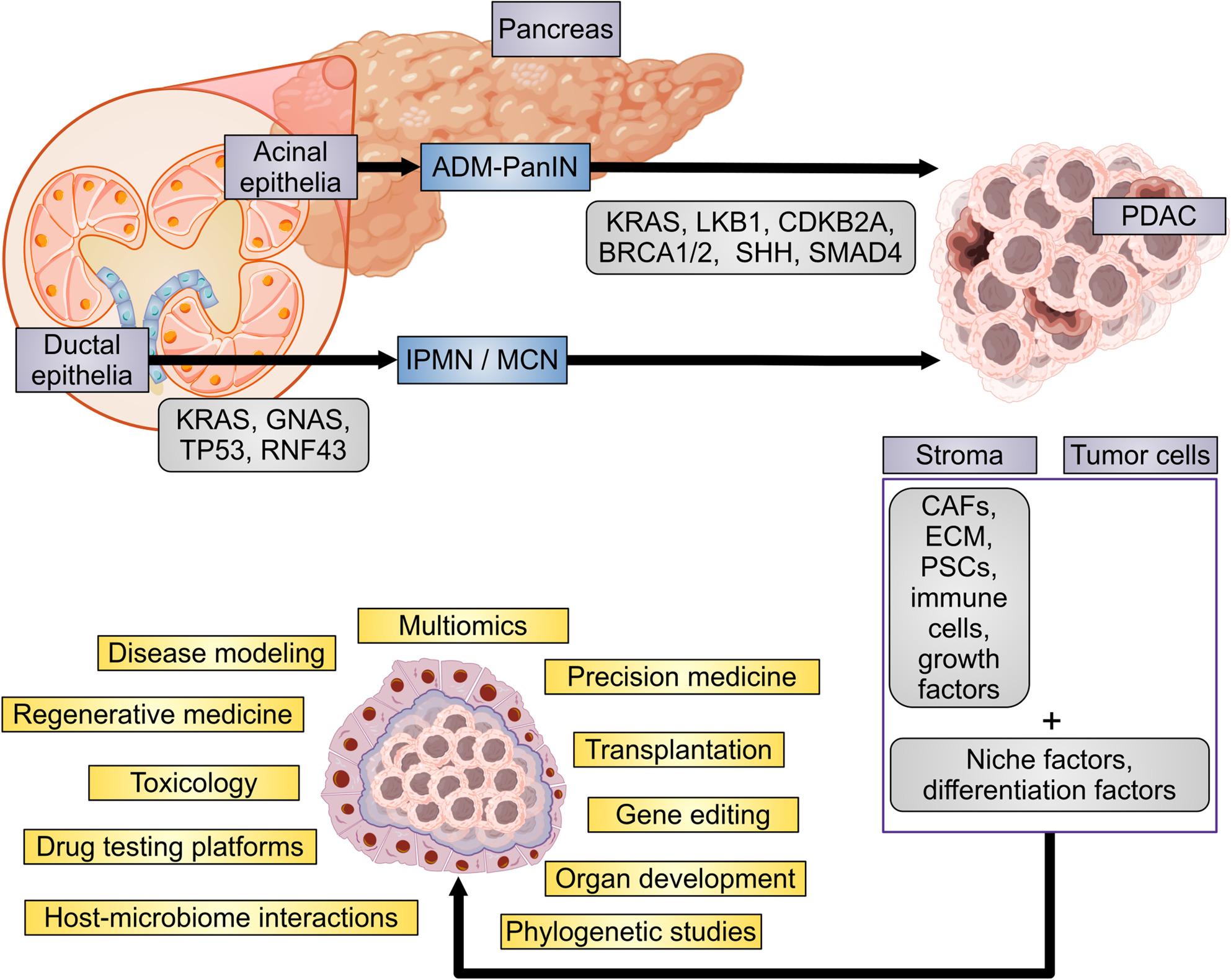

## Background

Pancreatic cancer is one of the most aggressive and lethal malignancies, often diagnosed at advanced stages [1]. While survival rates for most tumors have improved, pancreatic cancer mortality remains consistently high. It is on track to become the second most frequent cause of cancer death within the next decade [2].

More than 95% of pancreatic cancers arise from the exocrine compartment, comprised of acinar and ductal cells [3]. Pancreatic cancers can be classified into four types: (i) pancreatic ductal adenocarcinoma (PDAC; >90% of cases [4], (ii) acinar cell carcinoma (ACC), (iii) adenosquamous carcinoma (ASC), and (iv) anaplastic carcinoma of the pancreas (ACP) [5]. Only about 12% of patients with unresectable disease are predicted to survive 5 years [2].

The epidemiological risk factors for PDAC comprise factors such as type 2 diabetes mellitus, chronic pancreatitis, high-fat diets, non-O blood group, obesity, and life-style habits including alcohol and smoking [6]. In addition, several frequent mutations in oncogenes and the dysregulation of suppressor genes like Kirsten rat sarcoma oncogene (KRAS), cyclin-dependent kinase inhibitor 2 A gene (CDKN2A), tumor protein 53 gene (TP53), and small mothers against decapentaplegic homolog 4 gene (SMAD4) appear to be involved in PDAC development [3].

The bad prognosis of PDAC is mainly due to late diagnosis, no specific PDAC-related symptoms, high genetic heterogeneity, lack of efficient treatment options, and limited surgical procedures [7, 8]. However, there are biomarkers like apolipoprotein A1 (ApoA1), cancer-antigen 125 (CA-125), carbohydrate antigen 19 − 9 (CA19-9), sialic acid-containing carbohydrate antigen 242 (CA242), carcinoembryonic antigen (CEA), apolipoprotein A2 (ApoA2), and transthyretin (TTR), which are suitable for PDAC early diagnosis [9, 10]. Among these, CA19-9 is the most commonly used biomarker from serum with the highest sensitivity (75.45%) whereas CA242 (83%) has the highest specificity for pancreatic cancer [9].

The only cure is complete surgical resection, but most patients are not eligible for surgery due to locally advanced or metastatic tumor disease [11]. Recently, however, it has become possible to generate both malignant and non-malignant pancreatic carcinoma organoids as meaningful 3D culture models and also to reduce animal testing. Although these techniques are still costly and labor-intensive, they significantly expand the repertoire of preclinical research methods. The aim of this review is therefore to summarize the current knowledge on the generation and cultivation of 3D cell culture models for pancreatic carcinoma to highlight the special features of pancreatic cancer, such as its extremely poor oxygen supply and to discuss their significance for preclinical research. Therefore, this review is organized around the major domains tumor biology, tumor microenvironment (TME), culture systems, and clinical applications that are important to understand the value but also the limitations of pancreatic cancer organoid research. Hence, this review aims at stimulating further research in this extremely important field and reading this review serves the purpose of facilitating cutting-edge research, comparing protocols, and shedding light on the use of these powerful tools from different perspectives, ultimately enabling the testing and, in perspective, the translational application of new therapeutic approaches.

### The tumorigenesis of PDAC

The tumorigenesis of pancreatic ductal adenocarcinoma (PDAC) is a stepwise evolutionary process. We first describe the development from well-characterized precursor lesions before we discuss the genetic ladder and the unique TME in more detail.

In contrast to other organs of the gastrointestinal tract, the pancreas comprising cells of exocrine (acinar), epithelial (ductal), and endocrine (α, β, δ, ε) origin, appears to be deficient of a defined stem cell compartment [1]. PDAC contains both ductal and acinar cells [12]. There is a rising consensus, that two major transcriptomic epithelial subtypes of PDAC exist: The basal-like squamous or quasi-mesenchymal and the classical pancreatic progenitor subtype. Tumors with a basal-like subtype associate to more dedifferentiated and advanced tumors with increased cytokeratin levels and poor prognosis [4]. The classical subtype shows expression of genes distinctive for pancreatic progenitor cells, is characterized by ductal differentiation markers, and correlates with better outcome [4, 13].

**Fig. 1:**
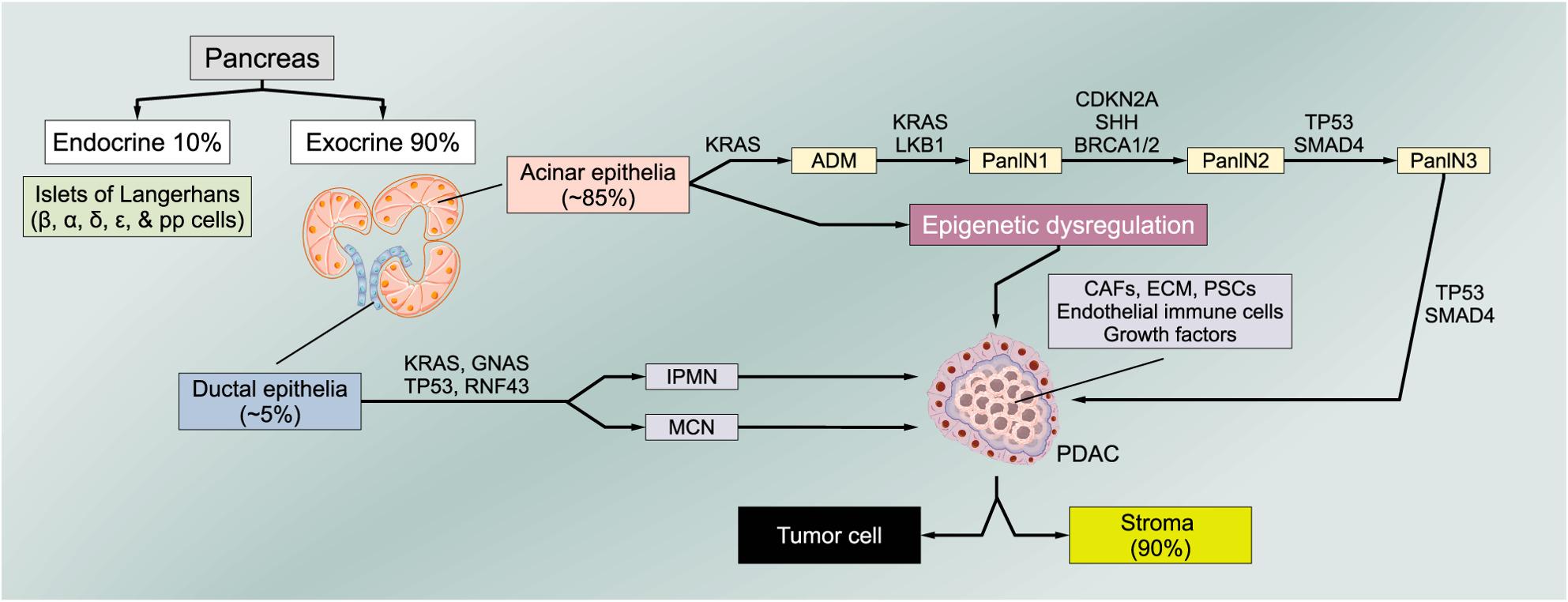
PDAC carcinogenesis. The progressive development of PanIN evolving towards PDAC is considered as the initial step in PDAC development followed by sequential progression involving mutations in several tumor suppressor genes (12). In this process, the gene encoding the proto-oncogenic GTPase KRAS as well as several tumor suppressor genes, including TP53, CDKN2A, and SMAD4, exhibit the most frequent alterations and/or mutations in PDAC (1). In addition, the transcriptomic landscape is influenced by epigenetic alterations. PDAC consists of tumor cells, and the stroma (represents up to 90% of the tumor volume), composed of CAFs, ECM, PSCs, endothelial cells, immune cells, and various growth factors. All components of the stroma seem to be required in the tumor organoid culture to closely model PDAC ex vivo (7). Abbreviations: CAF, cancer-associated fibroblast; CDKN2A, cyclin-dependent kinase inhibitor 2A; ECM, extracellular matrix; KRAS, Kirsten rat sarcoma oncogene; PanIN, pancreatic intraepithelial neoplasia; PDAC, pancreatic ductal adenocarcinoma; PSC, pancreatic stellate cell; SMAD4, small mothers against decapentaplegic homologue 4; TP53, tumor suppressor protein 53

PDAC is preceded by four subtypes of preneoplastic precursor lesions: (i) the intraductal papillary mucinous neoplasia (IPMN), (ii) pancreatic mucinous cystic neoplasm (MCN), (iii) intraductal tubular papillary neoplasm (ITPN), and (iv) pancreatic intraepithelial neoplasia (PanIN) [14]. The most frequently observed and most important precursor lesion of PDAC is the PanIN [15], a microscopic lesion that occur in the small pancreatic ducts, which cannot be observed on abdominal imaging scans [2, 8]. The noninvasive PanIN lesions were formerly classified into two stages: low-grade (PanIN-1 and PanIN-2) and high-grade (PanIN-3) [16]. A smaller proportion of PDACs (< 10%) arise from IPMNs, macrocystic lesions, that involve the pancreatic ductal system and differ from the least common mucinous cystic neoplasms, which do not involve the ductal system and have a characteristic ovarian-type stroma [8]. In a process called acinar-to-ductal metaplasia (ADM), acinar cells transdifferentiate to more epithelial (ductal-like) phenotypes resulting in a progressive development of PanINs towards PDAC. This transformation is generally considered as the initial step in PDAC development followed by sequential progression involving mutations in several tumor suppressor genes [12] (Fig. 1). The ADM stimulates the differentiation of pancreatic stellate cells (PSCs) in activated fibroblasts. Activated cancer-associated fibroblasts (CAFs) express alpha-smooth muscle actin (α-SMA), secrete extracellular matrix (ECM) components (collagen, fibronectin, laminin, and hyaluronic acid), inflammatory cytokines, growth factors and promote cancer development by releasing transforming growth factor beta 1 (TGF-β1), which prolongs the expression of oncogenic MYC by new PDAC cells. In the stage of the in situ PDAC, CAFs, found only in the invasive front, promote desmoplasia and tumor growth, at the expense of nearby pancreatic acini that proceed towards atrophy. Temporarily, cancer cells induce angiogenesis by liberating angiogenic factors like vascular endothelial growth factor A (VEGF-A) and tumor necrosis factor alpha (TNF-α), allowing interactions with surrounding cells as CAFs, pericytes, and endothelial cells. Cross-talk between the endothelial cells of the newly formed vessels and cancer-inducing cells favor cancer expansion by promoting the latter’s maintenance and growth. Moreover, the progression towards PDAC is initiated by cytotoxic T-lymphocytes (CTLs), stimulated by TGF-β, and marked by an increased presence of immune-suppressing cells in the TME, like regulatory T cells and M2 macrophages, other than the expansion of PSCs.

Several driver genes such as the proto-oncogenic GTPase KRAS as well as tumor suppressor genes, including TP53, CDKN2A, and SMAD4 were identified together with many additional mutations such as ring finger protein 43 (RNF43), guanine nucleotide binding protein, and alpha stimulating activity polypeptide (GNAS), generating an extremely high tumoral heterogeneity [17]. Whereas KRAS wild-type tumors harbored alterations in other oncogenic drivers, including GNAS, BRAF, catenin beta 1 (CTNNB1), and additional RAS pathway genes [17].

In addition to genetic alterations, the transcriptomic landscape is also influenced by epigenetic alterations (Fig. 1).Tumor suppressor genes have been described to be repressed, oncogenes upregulated, and epigenetic (re-)programing is fundamentally linked to tumor progression and metastasis formation [1].

### The PDAC tumor microenvironment

The PDAC-TME is an altered stroma localized at the interface between the tumor and the healthy parenchyma of the organ, playing a crucial role in tumor development, metastatic spread, immune escape, and chemoresistance [8]. It is composed of dense ECM, the vascular system, CAFs, and immune cells associated with a multitude of cytokines, growth factors, and ECM-metabolizing enzymes which translate into a dense tumor mesenchyme to provide an environment with limited anti-tumor immunity [18–20]. The matrix deposition results in increased interstitial pressure, compressing the vessels in the tumor. The deposition of ECM components appears to be positively influenced by the tumor itself through various ways such as the expression of missense mutations of TP53 in pancreatic cancer cells related with increased ECM production by CAFs [8].

During cancer progression, the tissue becomes less vascularized, generating an isolated hypoxic environment, which contributes to further disease progression, immuno-escape, and chemoresistance [8]. In the crosstalk between PDAC cells and TME, both Hedgehog, by acting via paracrine pathways, and TGF-β, synthesized by T-Lymphocytes, operating through autocrine and paracrine pathways, represent the main factors [8]. The TME represents a barrier to pharmacological intervention, increases the tumor progression, angiogenesis, and stromal formation [9]. Another characteristic of the TME is hypoxia, which affects the activity of various molecules and signaling pathways, compared with normoxia [21].

CAFs play a major role in PDAC progression. Human fine-needle aspiration (FNA) samples and mouse acinar cell organoids cocultured with CAFs revealed significant acino-ductal transdifferentiation and complex organoid development, and suggest that CAFs may induce ADM during PDAC onset. Parte et al. demonstrated that CAFs may profoundly induce complex crosstalk with acinar cell compartments via the laminin alpha 5 (LAMA5) / integrin alpha 4 (ITGA4) / signal transducer and activator of transcription 3 (STAT3) axis [22]. Therefore, the investigation of a LAMA5/ITGA4/STAT3 axis inhibitor could be a good approach to prevent ductal cell reprogramming and the development of ductal adenocarcinomas [22].

Hypoxia and fibrosis in the PDAC-TME protect the tumor mass from the patient’s immune system [8]. The limited immunogenicity and the poor T cell infiltration lead to insufficient antigen presentation, resulting in a weak or absent immune response and inadequate T cell trafficking and elicit a low efficacy of immune therapies in PDAC [20]. Few patients exhibit robust T cell infiltration in the tumor microenvironment often accompanied by extended survival time [23]. The clinical outcome of patients with PDAC is related to the composition of the TME [8]. While patients with poor prognosis showed signs of a more severe tumor-promoting infiltrate (M0 macrophages, memory B lymphocytes, and neutrophils), cancer-hostile immune cells (CD8+ and CD4+ cells, naive B lymphocytes, monocytes, plasma cells, and activated mast cells) were found in samples of patients with a better outcome [24].

While the role of epithelial-to-mesenchymal transition (EMT) in driving PDAC chemoresistance is widely recognized, Schuth et al. postulate a key role of CAF-driven induction of EMT in PDAC chemoresistance [25]. During coculture with CAFs, the authors observed increased proliferation and reduced chemotherapy-induced cell death in PDAC organoids [25]. Depletion of tumor stroma resulted in more aggressive tumors and reduced survival in PDAC patients [25]. Results from single-cell RNA sequencing (scRNA-seq) analyses identified several potential interactions involving the cancer stem cell marker CD44 with ligands secreted by CAFs such as hepatocyte growth factor (HGF), heparin-binding epidermal growth factor (EGF)-like growth factor (HBEGF), fibroblast growth factor 2 (FGF2), and galectin-9 (lectin galactoside-binding soluble 9; LGALS9) [25]. High expression of CD44 has been associated with poor prognosis in PDAC and evidence from patient-derived xenografts shows that CD44 cells are the source of PDAC relapse after gemcitabine treatment, rendering CD44 a promising therapeutic target against recurrent disease [25].

### Therapeutic options in the treatment of PDAC

For most patients with PDAC, cytotoxic chemotherapy remains the mainstay of treatment [26]. Therapeutic options are based on their performance status and include gemcitabine/nab-paclitaxel or FOLFIRINOX (5-fluorouracil (5-FU), leucovorin, irinotecan, and oxaliplatin), radiotherapy, targeted therapy, immunotherapy, and combination regimes with and without resection [4, 27]. For patients with advanced-stage or metastatic disease, comprehensive genomic profiling has revealed several potentially useful alterations in small subsets of patients [26]. Novel treatment approaches consider pathway inhibition, alterations of the DNA repair-system, immunotherapy, cancer metabolism, and targeting of the TME [26].

In recent years, molecular targeted therapy for pancreatic cancer has been rapidly developed. Drugs including erlotinib, cetuximab, trastuzumab, bevacizumab, and sacituzumab govitecan, targeting epidermal growth factor receptor (EGFR), human epidermal growth factor receptor 2 (HER-2), VEGF and trophoblast cell-surface antigen 2 (Trop2) affect related pathways and the proliferation, apoptosis, metastasis, and invasion of tumor cells [27]. Novel pathway inhibitors, including agents attacking the RAS–RAF–MEK–ERK pathway, neurotrophic tyrosine receptor kinase (NTRK) fusions, anaplastic lymphoma kinase (ALK), and cyclin-dependent kinase 4/6 (CDK4/6) are investigated in several studies and some of them have recently entered clinical testing in patients with PDAC [26]. Poly (ADP-ribose) polymerase (PARP) inhibitors take advantage of defects in DNA repair mechanisms by preventing DNA damage repair (DDR), such as those found in patients with loss-of-function mutations in breast cancer susceptibility gene 1/2 (BRCA1/2) and partner and localizer of BRCA2 (PALB2), or those with tumors of a BRCAness phenotype [26]. Cancer cells with mutations that prevent homologous recombination repair via other pathways, such as loss-of-function mutations in BRCA1/2, are often exquisitely sensitive to PARP inhibitors [26].

Since the KRAS^G12D^ mutation is present in nearly half of PDAC, Mahadevan et al. investigated the effects of inhibiting the KRAS^G12D^ mutant protein with MRTX1133 on early and advanced PDAC and its influence on the TME. As a result, MRTX1133 reverses early PDAC growth, increases intratumoral CD8+ effector T cells, decreases myeloid infiltration and reprograms CAFs. MRTX1133 leads to regression of both established PanINs and advanced PDAC [28]. Other therapeutic approaches are studying the use of patient-derived tumor-infiltrating lymphocytes (TIL) in combination with autologous pancreatic cancer cell exposure for neoepitope generation.

Immunotherapy is still associated with a low success rates, due to the TME, enhancing immune escape of the tumor [9, 21]. The major hindrance is the fibrotic stroma, which prevents lymphocyte infiltration [29].

Analyses of large PDAC genomic datasets showed that only a subset of pancreatic cancers are immunologically active and due to relative low tumor mutation borders, treatment with immune-checkpoint inhibitors (ICIs) showed a limited response [30]. ICIs were only approved for the small subset of PDAC tumors with high microsatellite instability (1–2% of all cases) [1, 9]. Nontheless, the implementation of immunotherapy for pancreatic cancer, including ICIs, vaccination, and adoptive T cell transfer, has come into focus, after preclinical research showed some promise [9]. Vaccinations and adoptive T cell transfer both increase the specificity of T cells to attack cancer cells [9]. Recent therapy options for pancreatic cancer aim at reducing the immunosuppressive TME, including the use of the most advanced stromal modulator, pegvorhyaluronidase alfa (PEGPH20), focal adhesion kinase (FAK) inhibitors, connective tissue growth factor (CTGF) inhibitors, Bruton tyrosine kinase (BTK) inhibitors, chimeric antigen receptor (CAR)-T lymphocytes against HER2, fibroblast activation protein (FAP), CEA, melastatine (MLSN), prostate stem cell antigen (PSCA), or CD133 [8, 26]. PEGPH20 disassembled stromal proteins, increased intratumoral blood flow, and improved progression free survival in a phase II trial, when added to chemotherapy [8].

The most common cause of PDAC chemoresistance is due to the ability of cancer cells to spread out and fill the pancreatic parenchyma, exchanging nutrients, substrates, and even genetic material with cells from the surrounding TME [8]. Additionally, a combination of hypoxia, decreased pH, and significant interstitial fluid pressure contributes to tumor survival and downregulation of antitumor immune cells [9]. It is well known that chemotherapeutic treatment induce plasticity of PDAC cancer cells undergoing transcriptional subtype switching, e.g. from Basal-like-B subtype to Classical-A subtype adapt to the medication [31]. Further investigations are required to understand how the plasticity-emerged subtypes impact prediction of treatment response [32].

However, combinations of immunotherapy, chemotherapy, and radiation therapy have proven to be the most effective method in the treatment of pancreatic cancer. Despite therapeutic intervention, median overall survival is 6.7–11.1 months (progression free survival (PFS) = 3.3–6.4 months) for advanced disease, compared to 25–28 months (PFS = 13.1–13.9 months) in surgically resected patients.

### The influence of different oxygen levels on PDAC progression

Various studies reveal that median oxygen levels vary between tumor types. It is well-known that many prostate and pancreatic tumors are profoundly hypoxic [33]. Among the solid tumors, pancreatic cancer is the most hypoxic and it has long been recognized that tumors with areas of hypoxia are the most aggressive and difficult tumors to treat [34]. Compared to healthy pancreatic tissue with an oxygen pressure of 30–50 mmHg, which is decreased to 2.5 mmHg in solid tumors, PDAC is considered severely hypoxic, with about 0.7% oxygen content, whereby hypoxic sites are heterogeneously distributed throughout the tumor tissue [35]. The hypoxic environment is based on the imbalance of secreted angiogenic activators and inhibitors as well as the decreased oxygen diffusion (~ 200 µmol/L) [6]. These hypoxic areas contribute to malignant progression, resistance to chemotherapy, radiotherapy, metastasis, and poor patient prognosis [6, 36]. This indicates that cancer cells might adapt to the stressed condition, overcome it, and gain an advantage for survival and growth [37]. Pancreatic tumor cells might be particularly hypoxia tolerant because they survive oxygen levels ≥ 19- fold lower than those found in normal pancreatic tissue [38].

Central to this adaption is the hypoxia-inducible factor (HIF)-regulated signaling network. Before discussing a promising therapeutic approach, we illustrate the critical role of the HIF signaling axis in driving chemoresistance and the EMT.

Particularly, HIFs play a pivotal role in the adaptation of tumor cells to hypoxic and nutrient-deprived conditions by upregulating the transcription of several pro-oncogenic genes [36]. The most extensively studied tumor response to hypoxia is through HIF-1α, whose levels rapidly increase during hypoxia [34]. The genes regulated by HIF-1α encode proteins involved in erythropoiesis, glycolysis, promotion of cell survival, angiogenesis, and inhibition of apoptosis as well as immune cell activation [37]. HIF-1α also targets fascin, an overexpressed protein in pancreatic cancer and enhances scattering, motility, and invasiveness of cancer cells [6]. Furthermore, HIF-1α has been shown to induce the conversion of non-stem pancreatic cells into pancreatic cancer stem-like cells, which are responsible for tumor formation, progression, drug resistance, metastasis, and recurrence. Tumors, that express high levels of cancer stem-like cell markers like CD44 exhibit a longer average survival rate. Thus, the treatment of pancreatic cancer cell lines with a CD44 antibody downregulates the stem cell self-renewal genes sex determining region Y-box 2 (Sox-2) and Nanog, reduced expression protein 1 (Rex-1) and STAT3-mediated cell proliferation, ultimately leading to decreased metastasis and tumor growth in mice [6].

Investigations have illustrated that increased HIF-1 activity increases tumor growth, vascularization, and glucose metabolism, whereas loss of HIF-1 activity contribute to a significant reduction of these responses. Consequently, it is concluded, that increased HIF-1α levels are a marker of aggressive clinical disease associated with poor patient prognosis and treatment failure in different cancers [39, 40]. Except intratumoral hypoxia, several mechanisms have been reported to contribute to HIF-1/2 signaling and regulation, including, low-molecular weight signaling molecules such as reactive oxygen species (ROS), cytokines, and growth factors, loss of tumor suppressor function, and oncogene gain of function [36]. However, utilizing the hypoxic tumor environment could be an attractive approach in the treatment of PDAC and makes the process of designing hypoxia-activated prodrugs (HAPs) or HIF-1/2 α inhibitors very promising.

### HIF regulation and its role in tumorigenesis and possible therapeutic strategies in the treatment of PDAC

HIF is a heterodimeric transcription factor, which consists of an O_2_-sensitive α (HIF-1α, HIF-2α, or HIF-3α) and an O_2_-insensitive, constitutively expressed HIF-1β subunit and controls cellular responses to hypoxia [41]. The HIF-α subunits are cytosolic and while HIF-1α is ubiquitously expressed at low levels in all tissues, HIF-2α and HIF-3α are expressed more tissue specific [42]. Conversely, the HIF-1β subunit is a constitutively active DNA binding protein that remains in the nucleus [42].

**Fig. 2:**
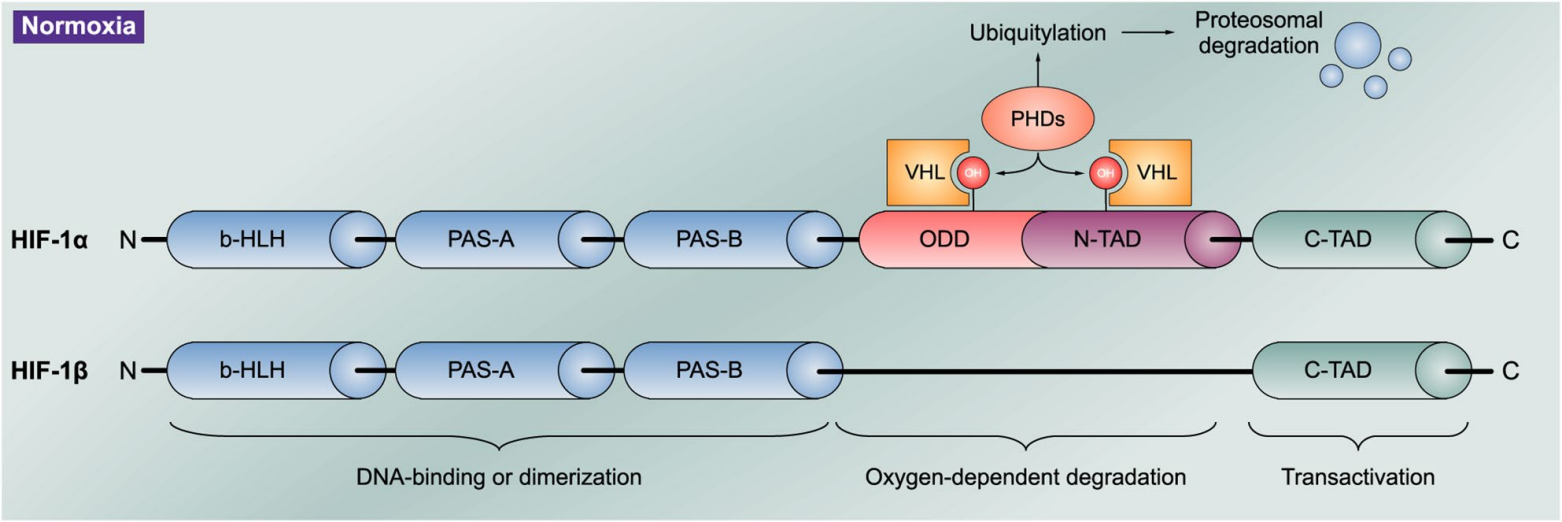
HIF-1α and HIF-1β structural schematic. The regulation of HIFs is mediated by several enzymes, including FIH, HIF-PHDs, and pVHL. HIFs consist of α (HIF-1α, HIF-2α, and HIF-3α) and β (HIF-1β) subunits, with the α-subunit acting as the main regulator of HIF transcriptional activity. Structurally, HIF-1α and HIF-2α contain an N-terminal bHLH domain and two PAS domains (PAS-A and PAS-B), which are required for DNA binding and dimerization with ARNT in response to hypoxia [44]. Both HIF-1α and HIF-2α consist of transcriptional activation domains at the N-terminus (N-TAD) and the C-terminus (C-TAD), activating the transcription of hypoxia-inducible genes, and playing a role in regulation by hydroxylation in normoxia [42]. Unlike HIF-1α and HIF-2α, HIF-3α lacks the C-TAD and is therefore considered an inhibitory HIF factor. Whereas HIF-1β is characterized by the absence of ODDD and N-TAD [45]. Under normoxic conditions, hydroxylation of specific proline residues on HIF-1α, HIF-2α, and HIF-3α and subsequent proteasomal degradation occur. This process involves the binding of pVHL and the initiation of ubiquitylation and results in obstructing the binding of coactivators CREB and histone acetyltransferase CBP-p300 and inhibiting HIF function. In hypoxic conditions, stabilization and accumulation of active HIF-α–HIF-1β complexes arises, leading to the subsequent binding to hypoxia-response elements in target genes and the induction of HIF target genes [44]. Abbreviations: ARNT, aryl hydrocarbon receptor nuclear translocator; bHLH, basic helix-loop-helix; CBP-p300, CREB-binding protein E1A binding protein p300; CREB, cyclic adenosine monophosphate response element binding protein; FIH, factor inhibiting HIF; HIF, hypoxia-inducible factor; HIF-PHD, hypoxia-inducible factor prolyl hydroxylase domain; ODDD, oxygen-dependent degradation domain; PAS, Per-Arnt-Sim; pVHL, von Hippel–Lindau protein; TAD, transcriptional activation domain

While the two Per-Arnt-Sim domains (PAS-A and PAS-B) are necessary for the heterodimerization between HIF-α and HIF-1β, both HIF-1β and HIF-α subunits have an oxygen-dependent degradation (ODD) domain that mediates hydroxylation of two proline residues and the acetylation of a lysine followed by proteasomal degradation. In addition, the HIF subunits contain two transcriptional activation domains: the N-terminal transactivation domain (N-TAD) within the ODD domain and the C-terminal transactivation domain (C-TAD). The proline residues are conserved in HIF-1/2α subunits [43] (Fig. 2).

**Fig. 3:**
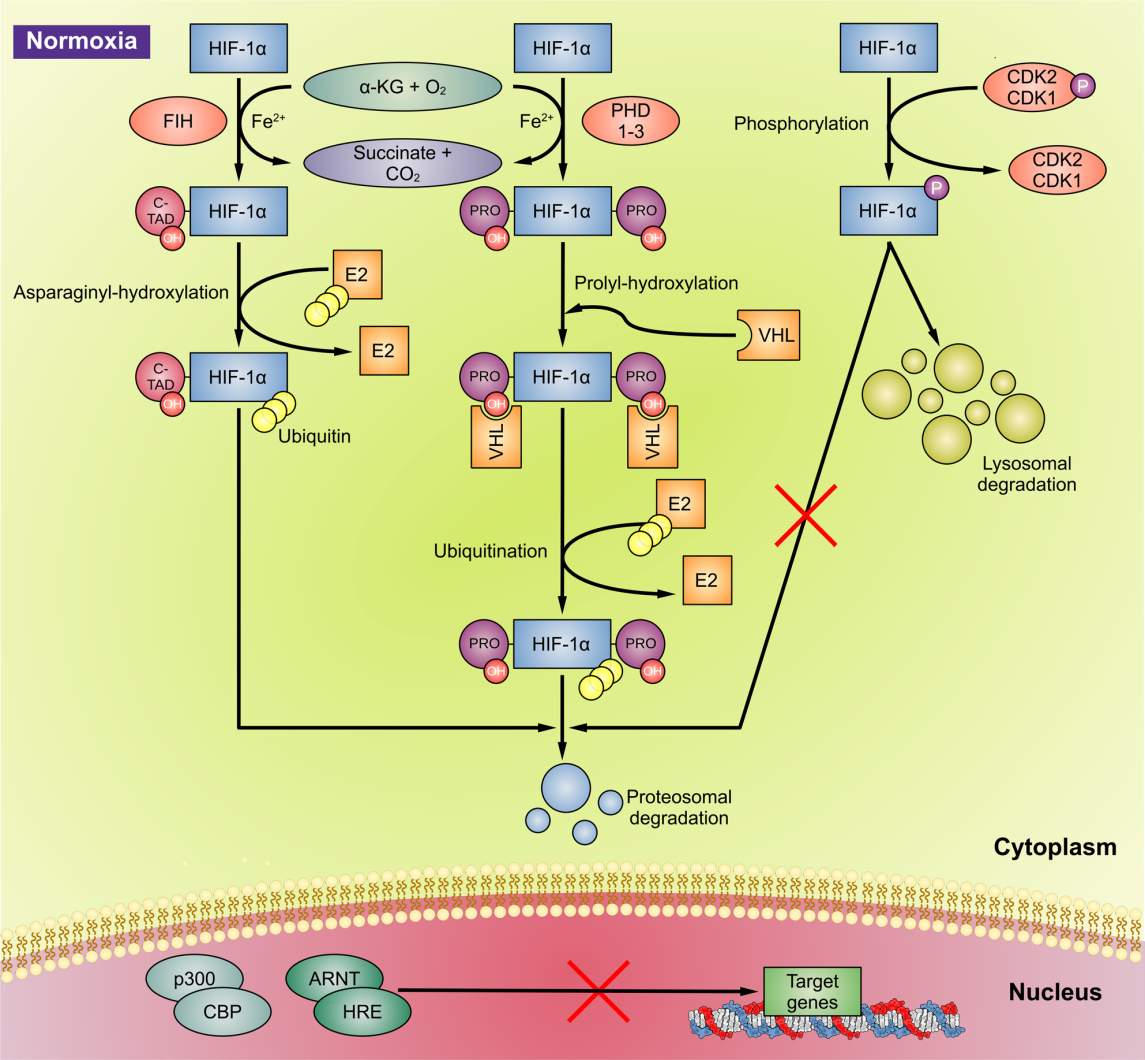
HIF regulation in normoxia. Under normoxia, HIF-1α activity is regulated by different pathways (prolyl hydroxylation, asparaginyl hydroxylation, phosphorylation, and ubiquitination) and enzymes (PHD, FIH, and CDK1/2) to promote HIF degradation. The hydroxylation of HIF-1α, catalyzed by PHD, allows binding of VHL, which recruits an ubiquitin ligase complex, leading to HIF-α ubiquitination and proteasomal degradation. FIH, an asparagine hydroxylase, hydroxylates HIF-1α, inhibiting the transcription (nucleus) and translation (cytoplasm) of HIF-1α. The activity of HIF-1α is also controlled by CDK2. CDK2 phosphorylates HIF-1α, inhibits proteasomal degradation, and activates lysosomal degradation [42]. Abbreviations: α-KG, α-ketoglutarate; ARNT, aryl hydrocarbon receptor nuclear translocator; CDK1/2, cyclin-dependent kinase 1/2; CREB, cyclic adenosine monophosphate response element binding protein; FIH, factor inhibiting HIF; HIF-1α, hypoxia-inducible factor-1α; HRE, hypoxia response element; PHD, propyl hydroxylase domain enzyme; PRO, proline; VHL, von Hippel-Lindau tumor suppressor protein

Under normal oxygen tension, HIF-1 activity is usually suppressed due to the rapid, oxygen-dependent degradation of the HIF-1α subunit [46]. In normoxia, HIF-α activity is regulated by several degradation pathways and regulatory proteins. Possible degradation pathways include hydroxylation, ubiquitination, SUMOylation (conjugation of small ubiquitin-related modifier protein), S-nitrosylation, asparagine hydroxylation, and phosphorylation (Fig. 3).

Among the regulatory proteins, both the prolyl hydroxylase domain (PHD) enzyme and factor-inhibiting hypoxia-inducible factor (FIH) hydroxylate HIF. While PHD hydroxylates HIF at proline residues and enable the recruitment of von Hippel-Lindau tumor suppressor protein-E3 ubiquitin ligase complex (pVHL-E3), FIH hydroxylates HIF in the C-TAD, the binding sites of co-transactivators p300/CBP (E1A binding protein p300/cAMP response element-binding protein (CREB)-binding protein) that promote transcription of HIF target genes [43]. Ultimately, both pathways lead to degradation in the presence of oxygen as co-substrate.

**Fig. 4:**
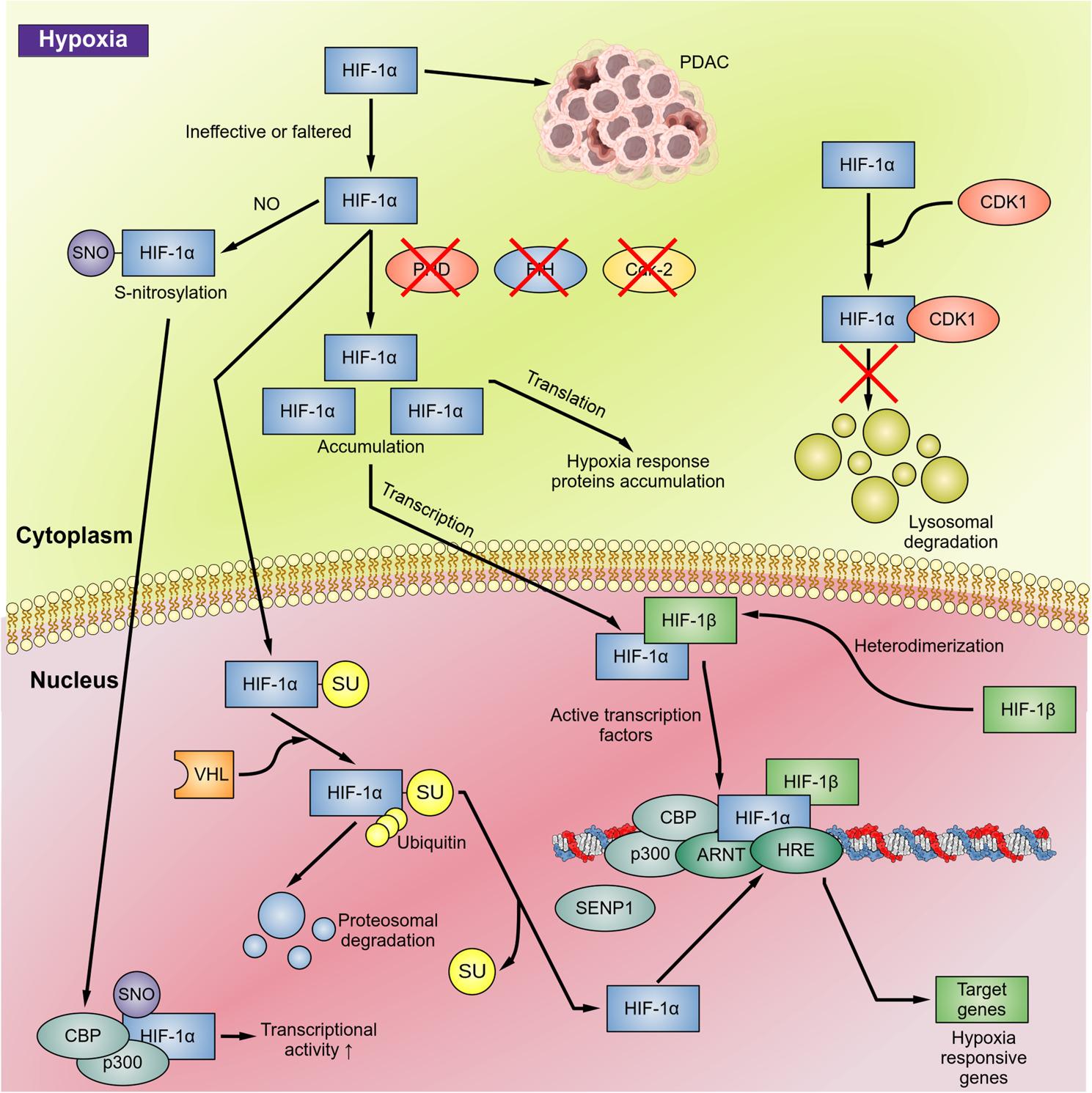
HIF regulation in hypoxia. In hypoxia, HIF-1α subunits accumulate, translocate to the nucleus, and activate target gene transcription. As a result of hypoxia, the inhibition of HIF-1α hydroxylation (catalyzed by PHD and FIH), and the inhibition of CDK2 as well as the HIF-1α S-nitrosylation lead to HIF-1α stabilization and accumulation, enhancing transcriptional activity of HIF-1α. In addition, accumulated HIF-1α binds to CDK1 and inhibits the lysosomal degradation. SUMOylation provides an alternative signal for VHL- and ubiquitin-dependent degradation, while in hypoxia, SENP1 leads to HIF-1α stabilization and increased transcriptional activity [42]. In the nucleus, stabilized HIF-1α subunits dimerize with HIF-1β subunits and ARNT, recruiting CBP-p300, and enabling interaction with HRE in a wide range of gene promoters in the nucleus. The resulting pattern of transcription controls cellular responses to hypoxia such as angiogenesis and metabolic reprogramming and is able to upregulate the expression of multiple genes that contribute to cancer progression due to survival, metastasis, and invasion [48]. Abbreviations: ARNT, aryl hydrocarbon receptor nuclear translator protein; CBP-p300, CREB-binding protein E1A binding protein p300; CDK, cyclin dependent kinase; CREB, cAMP response element binding protein; FIH, factor inhibiting HIF; HIF-1α, hypoxia-inducible factor-1α; HRE, hypoxia response element; PHD, propyl hydroxylase domain enzyme; SENP1, Sentrin-specific protease 1; SNO, S-nitrosylation; SUMO, small ubiquitin-like modifier; VHL, von Hippel-Lindau tumor suppressor protein.

In hypoxia, hydroxylation cannot occur, causing HIF-1α and HIF-2α stabilization, accumulation, dimerization with HIF-1β, and translocation to the nucleus [41, 43] (Fig. 4). Hypoxic conditions foster the interaction of the HIF C-TAD domain with coactivators such as p300/CBP and allow HIF to recruit a larger transcriptional device to hypoxia responsive genes [43]. In the nucleus, HIF-α subunits dimerize with aryl hydrocarbon receptor nuclear translocator (ARNT) protein element and the HIF/ARNT heterodimer recruits p300/CBP, forming a complex that binds to the hypoxia response elements (HRE) in promoter regions to activate target gene transcription [47]. Consequently, HIF can translocate in the nucleus to initiate transcription or can remain in the cytoplasm to initiate translation of hypoxia-responsive proteins (Fig. 4). HIF-dysregulation by PHDs or FIH (factor inhibiting HIF) may lead to cancer.

An additional degradation pathway of HIF-1α is the SUMOylation, catalyzed by SUMO-specific E1, E2, and E3s and reversed by Sentrin/SUMO-specific proteases (SENPs). Hypoxia induces nuclear translocation and SUMOylation of HIF-1α, which provides an alternative signal for VHL- and ubiquitin-dependent degradation. SENP1 stabilizes HIF-1α by removing the alternative VHL-binding signal and contributes to HIF-1α stabilization and increased transcriptional activity [49].

As a result of hypoxia, the nitric oxide (NO) levels increase, causing HIF-1α S-nitrosylation, which in turn promotes HIF-1α binding to transcriptional co-factors, such as p300/CBP, enhancing its transcriptional activity [46]. The recognition of the role of NO in the up-regulation of HIF-1α during cancer therapy suggests a promising strategy to improve current therapy: the use of nitric oxide synthase (NOS) inhibitors in conjunction with conventional radiation and chemotherapy modalities [46]. Li et al. established the importance of nitric oxide-mediated S-nitrosylation in regulating the stability of HIF-1α. They indicated that S-nitrosylation of Cys533 (murine equivalent of human Cys520) in HIF-1α is directly responsible for radiation-induced HIF-1α stabilization in tumors. They also suggested that modulating HIF-1α activation through NOS inhibitors may be a promising strategy for therapeutic development in a variety of diseases such as cancer and inflammatory diseases where it has been assured that both NO and HIF-1α play prominent roles [46].

HIF-1α is also regulated by the cell-cycle regulator protein cyclin-dependent kinase 2 (CDK2). Under normoxic conditions, CDK2 phosphorylates HIF-1α, inhibiting proteasomal degradation and activating lysosomal degradation as a secondary mechanism of HIF regulation in normoxia [50]. In hypoxia, CDK2 is inhibited, leading to accumulation of HIF-1α to initiate cellular responses. The cell cycle regulator protein CDK1 also phosphorylates HIF-1α and promotes lysosomal degradation in normoxia. Whereas in hypoxia, accumulated HIF-1α binds to CDK1 and inhibits the lysosomal degradation pathway [50].

In addition to these regulatory proteins, both non-coding RNAs and hypoxia-responsive long non-coding RNA (HRL) modify the mediation of cellular response to hypoxia. While the non-coding RNAs, micro-RNA-429 and micro-RNA-210, directly bind to the HIF-1α gene and decrease the expression of HIF-1α and create a negative feedback loop of HIF-1α [42]. The HRLs, associated with increased tumorigenesis, ionizing radiation therapy resistance, and metastasis, create a positive feedback by stabilizing HIFs by disrupting the HIF-VHL interaction, resulting in HIF accumulation [51]. Disrupting the interaction between HIF-1α and p300 is a promising strategy to modulate the hypoxia response of tumor cells [52]. However, methods to identify and investigate pathways involved in tumorigenesis are desperately needed. In addition to using positron emission tomography radiotracers to track metabolism and hypoxia, diffusion-weighted magnetic resonance imaging qualifies as a patient-based non-invasive surrogate for tumor hypoxia in PDAC [53, 54]. Diffusion-weighted magnetic resonance imaging is well-suited for longitudinal monitoring of tumor hypoxia during the course of treatment [54]. Both approaches offer significant potential for designing new diagnostic and treatment approaches.

### Organoids as a promising approach in the early diagnosis and therapy of PDAC

**Fig. 5:**
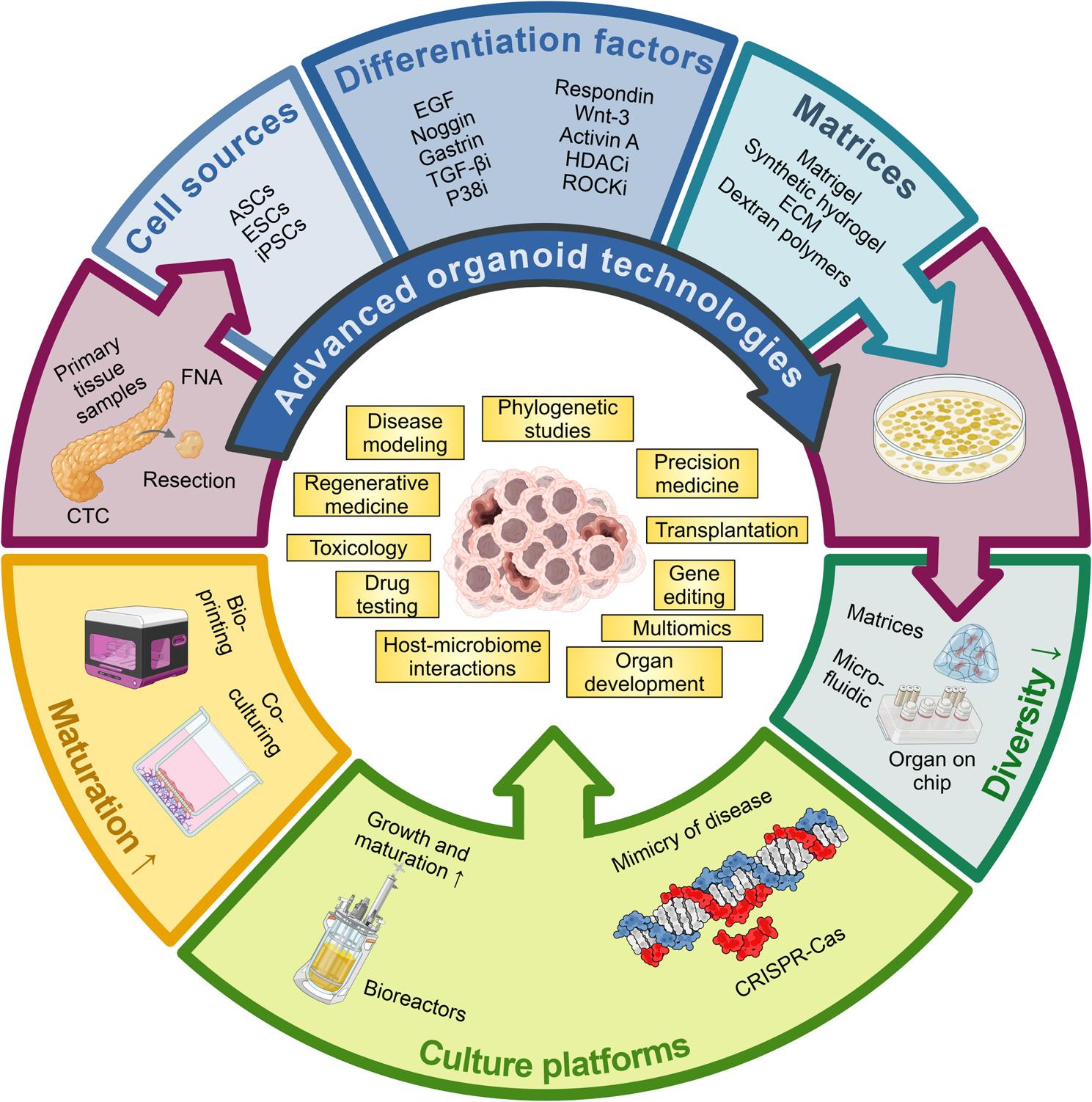
Generation and cultivation of organoids and their potential application areas. Organoids may be generated from various PDAC sources, including primary tumors, surgical resections, FNAs, CTCs, and metastatic lesions [8]. These models can be established using ESCs, iPSCs, or ASCs [56, 57]. The cultivation of organoids require stem cell niche factors, differentiation factors, and inhibitors, and the use of scaffold (biological or synthetic hydrogels, imitating the natural ECM) or scaffold-free techniques [58]. The integration of advanced cutting-edge techniques such as matrices, microfluid technology, CRISPR-Cas9 gene editing, bioreactors, coculture, and bioprinting improves the fidelity of disease models, advances organoid engineering, and optimizes biological functionality. The middle part illustrates the schematic representation of different application areas of the organoid technology, including disease modeling, multiomics, precision medicine, transplantation, gene editing, organ development, phylogenetic studies, host-microbiome interactions, drug testing platforms, toxicology, and regenerative medicine [58]. Abbreviations: ASCs, adult stem cells; CAF, cancer-associated fibroblast, Cas, CRISPR-associated protein; CRISPR, clustered regularly interspaced short palindromic repeats; CTC, circulating tumor cell; ECM, extra-cellular matrix; EGF, epidermal growth factor; ESC, embryonic stem cell; FNA, fine needle aspiration; HDACi, histone deacetylase inhibitor; iPSC, induced pluripotent stem cell; PDAC, pancreatic ductal adenocarcinoma; ROCKi, rho-associated, coiled-coil-containing protein kinase inhibitor; TGF-βi, transforming growth factor β inhibitor; Wnt, wingless/integrase-1. The figure contains icons from BioRender

Organoid technology offers new options in the fight against PDAC by optimizing both diagnosis and treatment options. Cancer organoids have become a widely accepted powerful tool in cancer research, imitating the distinctiveness of the organ and are supposed to mimic and render the organ specific functions and the organ´s cell type diversity [7, 55]. The application of organoids offers glimpses into pancreatic cancer progression, invasion, and heterogeneity. In addition to genome editing, transplantation, and oncogene identification, the organoids deliver a platform for drug and radiotherapy screening [55]. Due to the organoids appear to be genetically stable over serial passages, they are an ideal tool for PDAC modeling and drug testing (Fig. 5).

Drug screening for personalized medicine approaches is the preferred role of cancer organoid applications in cancer research to screen the most effective drugs and predict their therapeutic effects with the aim of entering clinical practice for guiding personalized therapy (Fig. 4) [7, 55]. Patient-derived organoids (PDOs) can predict response to neoadjuvant chemotherapy (NAT) in patients with pancreatic cancer. Longitudinal PDO generation derived from chemotherapy-naïve and post-NAT tissue are able to maintain dynamic chemotherapy sensitivity profiling to advance precision medicine in PDAC [59]. Yuan et al. revealed a correlation between the pathological response to NAT and PDO chemotherapy response, particularly oxaliplatin. They demonstrated the viability of a rapid PDO drug screening and generated data within seven days of tissue resection. The orthotopic transplantation of cancer organoids has been established in preclinical models. While sequencing analysis is commonly used to identify type-specific differentially expressed genes, RNA sequencing analysis and whole-exome sequencing are performed to identify cancer-related oncogenes [55]. Moreover, the clustered regularly interspaced short palindromic repeats (CRISPR)-Cas9 technology can be used in genome editing to achieve fast and efficient gene knock-in in human wild-type organoids [55].

### Organoid culture

Various methods have been developed for the establishment and cultivation of organoids, each with their own advantages and disadvantages. The following sections therefore aim to provide an overview of the most commonly used protocols, along with their respective potential and limitations.

The isolation and establishment of pancreatic organoids were first performed in mice [60]. Huch et al. used mouse pancreas duct fragments to activate wingless/integrase-1 (Wnt) signaling and to express leucine-rich repeat containing G protein-coupled receptor 5 (LGR5), which produced a cyst-like structure that can self-replicate [60]. Progressively, multiple methods were designed in humans to isolate and culture PDAC organoids to protect their long-term culture and cryopreservation [7]. The PDAC organotypic cultures can be derived from embryonic progenitors, adult-derived stem/progenitor cells (ASCs), tumor samples, differentiated from induced pluripotent stem cells (iPSC) or from different sources that recapitulate different characteristics [61] (Fig. 5). The cells of origin of PDAC (acinar or ductal cells) and their implication on the progression of PDAC is still being discussed. While acinar cells are refractory to in vitro culture as they rapidly undergo transformation into ductal-like cells [32], several studies employed organoids from human healthy ductal cells to investigate the PDAC tumorigenesis. The introduction of PDAC driver mutations in normal organoids via CRISPR-Cas9 or over expression vectors was used to study PDAC initiation and progression [62, 63]. Lee et al. used organoids derived from healthy CD133+ ductal cells to express mutant KRAS^G12V^, and deleted CDKN2A, TP53, and SMAD4 [62], Seino and colleagues employed ductal cells isolated from “normal-like” regions enclosed to tumor tissue [63]. In addition to the different methodology to introduce KRAS^G12V^ (overexpression vs. knock-in), the two studies also differ in the culture media used to establish the organoids, and the in vivo implantation sample (orthotopic vs. subcutaneous) [32]. An approach to overcome the limitation of cultivating both, acinar and ductal cells, consists in the differentiation of human PSCs (hPSCs) into acinus- and ductal-like exocrine organoids [32]. This differentiation step requires a sequential protocol in which ESCs are first differentiated into pancreatic progenitor-like cells and then into acinus or ductal-like cells [3]. Using this approach, Huang and colleagues recently implicate that PDAC driver mutations result in cell-lineage-specific phenotypes [32]. Pancreatic organoids are well-known for their complexity in establishing an ECM (Fig. 5), particularly for PDAC, in which the stroma is a key factor in tumor progression and drug applications [64]. Accordingly, there are many different protocols to culture human PDAC organoids through which different types of organoids are constructed (Table 1). All these methods can be classified based on the composition of the fetal bovine serum (FBS), on the type of scaffold used for cultivation, or the presence or absence of Wnt or R-spondin in the medium [7]. Generally, organoids require cell niche factors and ECM, which permit stem cells to express their differentiation and self-organization capacity [7]. The components and their concentration in the media depend on the specific cancer type [55].

**Table 1:** Cultivation of human pancreatic organoids derived from different starting sources/materials

Reference	Huang L et al. 2015 [3]	Boj SF et al. 2015 [65]	Tiriac H. et al. 2018 [4]	Raghavan S et al. 2021 [66]	Loomans et al. 2018 [67]	Koblas et al. 2019 [68]
Tissue of origin	Human primarytumor tissue	Human normal and malignant pancreatic tissues	Human primary tumor tissue	Human primary tumor tissue	Human adult islet-depleted pancreatic tissue	Human adult islet-depleted pancreatic tissue
Digestion	Collagenase (2 h) followed by trypsin (10–30 min)	Collagenase II in human complete medium at 37 °C (≤ 16 h) TrypLE (15 min) at 37 °C	Digestion medium at 37 °C with mild agitation (up to 1 h)	Collagenase XI at 37 °C (15 min) in human complete organoid medium	TrypLE Express (5 min) followed by filtering the cell suspension over a 40 μm cell strainer	Dispase solution at 37 °C (45 min)
3D-culture of organoids	Plating in Matrigel and cultured in PTOM, Medium replacement with POMM with 5% Matrigel after 8 days every 4 days	Plating in GFR Matrigel and cultured in human complete medium	Plated in Matrigel and cultured in human complete feeding medium	Plated in 3D GFR Matrigel	Plated in Matrigel	Plated in GFR Matrigel and cultured in 1 ml of expansion medium, Medium was changed every 1–3 days
Serial passaging of organoids	On day 16: organoids were treated with collagenase for 2 h, dissociated with trypsin for 10–30 min, collected, and reseeded in 3D culture	-	-	10 µM Y-27632 was included in the culture medium of newly initiated samples until the first medium exchange For propagation, organoids were dissociated with TrypLE Express, re-seeding into fresh Matrigel and culture medium	-	Passaged each 7 to 10 days, harvested from Matrigel using Dispase solution at 37 °C for 45 min and further dissociated by Accutase solution at 20 °C for 20 min, followed by trituration Dissociated organoids were transferred to a fresh Matrigel culture system
Cultivation time	5 passages	> 20 passages	≥ 5 passages	-	≥ 10 passages	≥ 20 passages
Composition of medium	PTOM: DMEM with 1% B27, 50 µg/ml ascorbic acid, 20 µg/ml insulin, 0.25 µg/ml hydrocortisone, 100 ng/ml FGF2, 100 nM all-trans retinoic acid and 10 µM Y-27632 POMM: PTOM (contains 1% B27 and 50 µg/ml ascorbic acid) with 5% Matrigel every 4 days PODM I: DMEM with 1% B27, 300 µM 2-phospho ascorbic acid, 100 ng/ml FGF7, 10 ng/ml hEGF, 1 µM A83-01, and 1 µM DBZ PODM II: DMEM with 1% B27, 300 µM 2-phospho ascorbic acid, 100 ng/ml FGF7, 10 ng/ml hEGF	Human complete medium: AdDMEM/F12 medium supplemented with HEPES 1x, Glutamax 1x, penicillin/streptomycin 1x, B27 1x, Primocin 1 mg/ml, N-acetyl-L-cysteine 1 mM, Wnt3a-conditioned medium (50% v/v), R-spondin-1-conditioned medium (10% v/v), Noggin-conditioned medium (10% v/v), or recombinant protein 0.1 µg/ml, hEGF, 50 ng/ml, Gastrin 10 nM, FGF10 100 ng/ml, Nicotinamide 10 mM, and A83-01 0.5 µM	Human complete feeding medium: advanced DMEM/F12, HEPES 10 mM, 1x Glutamax, A83-01 500 nM, hEGF 50 ng/mL, mNoggin 100 ng/mL, hFGF10 100 ng/mL, hGastrin I 0.01 µM, N-acetylcysteine 1.25 mM, Nicotinamide 10 mM, PGE2 1 µM, B27 supplement 1x, R-spondin-conditioned medium 10%, Afamin/Wnt3a-conditioned medium 50% Digestion medium: 1 mg/ml Collagenase XI, 10 µg/ml DNAse I, 10.5 µM Y-27632 in human complete medium	Human complete organoid medium: advanced DMEM/F12, HEPES 10 mM, 1x Glutamax, 500 nM A83-01, 50 ng/mL mEGF, 100 ng/mL mNoggin, 100 ng/mL hFGF10, 10 nM hGastrin I, 1.25 mM N-acetylcysteine, 10 mM Nicotinamide, 1x B27, R-spondin-1-conditioned medium 10%, Wnt3a-conditioned medium 50%, 100 U/ml penicillin/streptomycin, and 1x Primocin	Expansion medium: advanced DMEM/F12, HEPES 10 mM,1x Glutamax, 1% penicillin/streptomycin, 1x B27, 1x N-2, 1,25 µM N-acetylcysteine, 50 ng/ml hEGF, 10 nM Gastrin, 100 ng/ml FGF10, 10% R-spondin conditioned medium, 100 ng/ml Noggin or 10% Noggin conditioned medium, 500 nM TGFβ inhibitor (A83-01)	Expansion Medium: advanced DMEM/F12, B27 supplement (w/o vitamin A), 50 ng/mlrecombinant hEGF, 500 ng/ml R-spondin-1, 50 ng/ml recombinant hFGF10, 50 ng/ml recombinant hHGF, 100 ng/ml recombinant hNoggin, 1.25 mM N-acetylcysteine, 10 mM nicotinamide, PGE2 3 µM, CHIR99021 5 µM, SB-431,542 10 µM, trichostatin A 20 nM

*DBZ* dibenzazepine, *DMEM* Dulbecco’s Modified Eagle’s Medium, *hEGF* human epidermal growth factor, *FGF* fibroblastic growth factor, *GFR* growth factor-reduced, *HEPES* 4-(2-hydroxyethyl)-1-piperazineethanesulfonic acid, *HGF* hepatocyte growth factor, *PGE* prostoglandin E2, *PODM* pancreatic organoid differentiation medium, *POMM* pancreatic organoid maintenance medium, *PTOM* pancreatic progenitor and tumor organoid medium, *TGFβ* transforming growth factor beta, *Wnt *wingless/integrase-1

Most protocols have in common, that the organoids are obtained by an initial mechanical dissociation of the tissue, followed by an enzymatic digestion with collagenase/dispase and TrypLE or accutase treatment, and subsequently seeding the tissue suspension onto ECM [7]. The success rate depends not only on the quantitative and qualitative characteristics of the sample but also on the use of the Rho-associated, coiled-coil-containing protein kinase (ROCK) inhibitor, in all steps of the isolation procedure and for the first passage [7, 55]. The original stem cell niche organoid culture medium contains noggin and the mitogens EGF and R-spondin, that cause stem cell hyperplasia. The addition of the niche factors Wnt3a, EGF, noggin, R-spondin-1, nicotinamide, A83-01 (inhibitor of TGF-β type I receptor activin-like kinase 5 (ALK5)) are required for the establishment of long-term organoid cultures. For differentiated culture conditions the media are supplemented with indolatam, FGF-10, 2% FBS, B27, retinoid acid, cyclopamine, or notch inhibitor [69]. Several working groups adapted this protocol (Table 1). Modifications of the growth factors provided in the original intestinal organoid culture medium allowed the establishment of epithelial organoid cultures from several gastrointestinal organs, including the pancreas [69].

Boj et al. showed that in contrast to human non-malignant organoids, which require TGF-ß inhibitors (A83-1 and noggin), R-spondin1, Wnt3a, EGF, and prostaglandin E2 (PGE2) for propagation, human tumor-derived organoids tolerated the removal of certain growth factors [65]. Among the pathways associated with pancreas development, Huang et al. found that TGF-β and notch inhibition facilitated differentiation into ducts and acini, while Hedgehog inhibition and Wnt activation at stage II and III of induction diverted the developmental program away from the pancreatic lineage [3]. Several studies reveal the niche dependency (Wnt, R-spondin, and TGF-β) of organoid cultures is changing by cancer progression process and subtype (basal-like, classical), suggesting the possibility of cell selection within the culture conditions [70]. Shroyer et al. also reported on a selection bias of the tumor organoid system, caused by competition among different clones [71]. To minimize clonal selection and avert confounding drug treatment effects, the medium contains reduced growth factors including Wnt3A, R-spondin1, TGF-β receptor inhibitor, EGF, and noggin [55]. In addition, the culture media should be improved to stimulate organoid growth and long-term expansion while minimizing the effect of growth factors in the media on the behavior of organoids [55].

Some organoid-protocol components are subject to large variations and their production/preparation lingers a technical challenge. Such as the production and long-term conservation of recombinant, active stable Wnt for the preparation of Wnt-conditioned medium, based on mouse cells overexpressing and secreting Wnt into the medium, as a key factor of the majority of PDAC organoid culture media [7]. Wnt signaling plays a substantial role in the regulation of multiple types of adult stem cells and progenitors. In the adult pancreas, Wnt signaling is inactive, yet it is essential for its development during embryogenesis [60]. Due to the physical properties, the secretion and stabilization of active Wnt in cultured cells requires bovine serum and the very high batch-to-batch variation implies that some groups prefer commercially prepared medium [7].

Finally the purified stem cells are embedded in an ex vivo substitute for ECM, such as basement membrane extract (BME), Matrigel^®^, or extracellular matrix components, maintaining 3D aggregation and polarization of stem cells [32, 69]. Stem cell proliferation first resulted in cystic spheroids, which then formed crypt-like buddings that further developed into “mini-guts” with distinct crypt-villus compartmentalization within two weeks [69].

### Matrigel^®^

Many organoids have been cultured in Matrigel^®^, which is prepared from the secretion of Engelbreth-Holm-Swarm mouse sarcoma cells and enriched for ECM proteins. Matrigel^®^ is very complex and poorly defined; proteomic analysis shows that it contains more than 1800 proteins [72] and mainly comprises laminin, entactin, proteoglycans, and collagen IV. It primarily contributes to the cellular architecture of organoids [55]. This complexity makes it difficult to elucidate Matrigel^®^-specific factors governing organoid development; associated by too many variations of Matrigel^®^. Moreover, the mechanical properties of Matrigel^®^ samples like elastic modulus, pore size, stress relaxation, and creep are heterogeneous and can have large effects on cell, organoid, tissue, and organ development [73]. Finally, the fact that Matrigel^®^ is originated from mouse cells hampers its use in human clinical transplantation due to potential immunogenicity [73]. Matrigel^®^, which suffers from batch-to-batch variability and ill-defined composition, disfavors stromal cell propagation, and mostly fails to replicate the pathological ECM of human cancers [74]. Consequently, interdependencies between tumor cells and the microenvironment are inadequately modelled in this system [74]. There are different undefined types of materials, focusing on ECM derived from decellularized tissues, collagen, and other biomacromolecules derived from natural sources, and defined matrices, including synthetic polymer hydrogels and gel-forming recombinant proteins and peptides [74].

### Organoid culture in decellularized extracellular matrix scaffold

ECM plays an important developmental role by regulating cell behavior through structural and biochemical stimulation. Tissue-specific ECM, achieved through decellularization, has been suggested in several strategies for tissue and organ replacement [75].

The methods of decellularization used are dependent on the target tissue and cannot be generalized easily [73]. Decellularized ECM has been prepared from various pancreatic cell sources, such as animal and adult human pancreas. But the human pancreas presents major challenges in decellularization due to a higher lipid content compared to animal models. Sackett et al. illustrate that discarded human pancreases can be successfully decellularized, delipidated, and processed for development of 3D scaffold casts and hydrogels which maintain their macromolecules and are not toxic to the growth and differentiation of several types of cells. Therefore, they may have value in regenerative medicine applications [75].

The inclusion of a homogenization step in the decellularization protocol significantly improved lipid removal and gelation capability of the resulting ECM, which was capable of gelation at 37 °C in vitro and in vivo, and is cytocompatible with a variety of cell types and islet-like tissues in vitro [75].

The advantages of decellularized ECM are the fast recap of organ function and the unnecessary additional chemical modifications. However, the composition of ECM, containing over 300 ECM proteins and many more ECM-associated proteins, is complex and each of them has a different biological function and stiffness [76]. This complexity makes it difficult to investigate the influences of ECM on organoid behavior and development. Both the quantity and the quality of ECM is limited by the availability and health of the donor [73]. In addition, the physical properties of decellularized ECM are difficult to control and modify. Due to a large variability of decellularization protocols at removing cells or immunogenic species, distinct host immune responses and failure of implants in clinical trials can result [73].

### Organoid culture in synthetic hydrogels

Synthetic hydrogels are promising because their mechanical properties, functionality, and erosion rate can be controlled [73]. Due to the ability to control chemical as well as mechanical properties, it is possible to duplicate the heterogeneity in stiffness and composition found in organs. It also allows the generation of interfaces between materials similar to those found in the ECM and replicates essential elements of material microstructure; each of these controls has implications for organ function and disease [73]. Below et al. recapitulated the altered tissue stiffness, a hallmark of pancreatic cancer by adjusting the hydrogel properties to engage mechanosensing pathways and alter organoid growth [74]. Synthetic hydrogels can also be made responsive to external stimuli. Materials like thermoreversible hyaluronic acid-poly (N-isopropylacrylamide)-based hydrogel or light-sensitive polyvinyl alcohol matrices can be useful for future organoid studies. The use of synthetic hydrogels may also open up new avenues by altering the porosity of the scaffold on which the cells are grown. Studies showing an explicit connection between pore size and organoid differentiation are urgently needed [73]. Synthetic hydrogels also have readily tunable viscoelastic properties. The effects of viscoelastic properties on cell culture and behavior are complex and affect matrix remodeling, cell spreading, migration, differentiation, and consequently, organoid fate [73].

Synthetic scaffolds, such as the polyethylene glycol (PEG)-based hydrogel scaffolds, offer several advantages to cell- and tissue-derived matrices, including control over growth conditions [74]. Below et al. describe a fully synthetic hydrogel extracellular matrix designed to elicit key phenotypic traits of the pancreatic environment in culture. They revealed a functional role of laminin – integrin α3/α6 signaling in establishment and survival of pancreatic organoids. Furthermore, they evolve a model consisting of pancreatic stromal cells incorporated in hydrogel to recapitulate a pathologically remodeled tumor microenvironment for studies of normal and pancreatic cancer cells in vitro [74].

Advantageous in choosing synthetic polymers for organoid culture is their tractable variation in structure and properties and can be used to explore the effects of mechanical and chemical cues on cellular fate [73]. To date, some materials, including PEG and poly (lactic-co-glycolic) acid have been approved by the U.S. Food and Drug Administration (FDA) for use in human therapeutics [73].

Adversely, for the attachment of the cells, some synthetic hydrogels require the insertion of cell-binding peptides. In case of incorrectly spatially positioned cues or when non-matching bioactive compounds are used, cells will not be able to interact with the material and undergo cell death [77]. In addition, the toxicity of the by-products also limits the choice of polymers that can be used in cell culture [78]. Ultimately, synthetic hydrogels used as medical implants can trigger foreign body reactions and can indirectly influence ECM remodeling [79]. To further understand how the ECM remodeling can be influenced, the differential secretion of matrix metalloproteases was studied [80]. To avoid possible foreign reactions, it is required to construed alternative matrices such as peptide and recombinant protein gels for organoid culture.

### Organoid culture with gel-forming recombinant proteins

For pancreatic organoid culture, the application of gel-forming recombinant proteins has been investigated. Jin et al. demonstrated the importance of the ECM microenvironment in pancreatic organoid differentiation. They revealed that dissociated single cells from liver and pancreas lead up to morphologically distinct insulin-expressing colonies in methylcellulose-based media containing either Matrigel^®^ or laminin hydrogel [81]. Laminin hydrogel was shown to promote endocrine cell differentiation from adult pancreatic ductal progenitor-like cells in vitro [81].

The use of gel-forming recombinant proteins is characterized primarily by the low polydispersity, the possibility of adding chemical cues with exact definition, altering the chemical and mechanical properties of the gel independently and of programming degradation rates [73]. The recombinant proteins are molecularly well-defined and can be adjusted to stiffness, viscoelastic behavior, and chemical functionality [73].

However, there are also some disadvantages regarding the use recombinant proteins. Not all proteins can be recombinantly expressed and refolding and functionality of these proteins can be challenging [73]. Various studies reveal the immunogenicity of several recombinant proteins and self-assembling peptides [82]. Studies are ongoing to determine the specific components and properties of the pancreatic ECM in order to establish a useful scaffold for the growth and maintenance of stem organoids.

Because organoids only comprise the epithelial layer without the native microenvironment of the surrounding mesenchyme, immune cells, nervous system, or muscular layer, a number of protocols developed 3D-coculture with fibroblasts and immune cells to recapitulate cell-cell-interactions [7, 55]. Schuth et al. established 3D-cocultures of primary PDAC organoids and patient-matched CAFs to investigate the effect of the fibroblastic compartment on sensitivity to gemcitabine, 5-FU, and paclitaxel treatments using an image-based drug assay [25]. While the presence of CAFs was shown to be conducive for the organoid growth, the coculture with immune cells displays a significantly lower sensitivity to chemotherapies compared to tumor organoids without immune cells [7].

### Advaced organoid techniques

The constrains of conventional organoid cultures are characterized by the lack of precise spatiotemporal control of the microenvironment, vasculature, tissue-resident immune cells, and the interactions between different tissues and organs. Besides, 3D organoid culture systems are unable to replicate the microenvironment of the organ and lack the signaling that induce organogenesis [83]. To overcome these limitations of the classic 3D culture techniques, the combination with other innovative technologies, such as organ-on-a-chip, 3D bio-printing, and CRISPR-Cas9-mediated homology-independent organoid transgenesis (CRISPR-HOT) have allowed the development of more suitable cancer models to facilitate the development of organoid research [83].

To advance the classic organoid culture, the integration with “organ-on-a-chip” technology offers a promising concept by reproducing complex, integrated organ-level physiological and pathological responses by creating fluid flow, immune interactions, and interorgan communications to mimic organs or tissues [84]. Shirure et al. successfully demonstrated this integration by developing a tumor-on-a-chip microfluidic platform to investigate the progression and response to chemotherapy and antiangiogenic therapy in cell lines and PDOs [85]. Moreover, Du et al. revealed a strong correlation between the on-chip behavior of PDOs and their clinical metastatic potential through the development of novel vascularized PDOs-on-a-chip [86]. In addition, the organ-on-a-chip method enables dynamic imaging, molecular analyses with high spatiotemporal resolution, and can revolutionize many fields, including toxicology and development of pharmaceuticals that rely on animal testing and clinical trials [84]. It serves as an ideal platform for assessing the preclinical effectiveness of drugs and will facilitate the screening of personalized therapeutic targets in the future [86].

In conventional organoid cultures, oxygen gradients, found in solid tumors, are often missed. Across various organ systems, hypoxia-modulated organoid-on-chip platforms reveal how low-oxygen environments have been integrated into advanced 3D microphysiological systems, including bioelectronic, microfluidic, and vascularized chips, to mimic in vivo physiology and investigate organ-specific responses [87, 88]. Deipenbrock et al. developed a microfluid tumor-on-chip model to recapitulate the microenvironment of PDAC by simulating key processes such as vascular transport, immune cell polarization, and drug delivery. This model facilitates a deeper exploration of potential drug synergies, the incorporation of more complex multicellular structures by introducing additional immune and non-immune cell types, and the comprehensive study of the direct and indirect effects on the entire TME [89].

3D bioprinting is characterized by layer-by-layer of bioinks in a spatially defined manner to design viable 3D constructs [90]. The integration of organoids with 3D bioprinting can facilitate the development of an advanced cancer model with structures that are more cellspecific and well-separated properties that are more suited for the growth and maturation of organoids [90]. Several studies demonstrated that bioprinted organoids can accurately mimic in vivo conditions and allow the multiple scales of vasculate and even nervous and immune system to investigate tumorigenesis and the TME interactions for multiple tumor types and drug screening [83]. To increase effectiveness of in vitro models for disease biology, developing the complex TME and drug testing, principles of microfluidics and 3D bioprinting were integrated with co-culture techniques [90].

Another promising tool, applicable to the organoid field, is the CRISPR–Cas9 technology [91]. CRISPR-HOT is a new genetic tool that can be used to achieve fast and efficient gene knock-in in organoids representing different tissues. The focus here is on avoiding extensive cloning and it outperforms homology directed repair in achieving precise integration of exogenous DNA sequences into desired loci [91]. Finally, CRISPR–HOT is used for labeling specific genes in human organoids, visualizing subcellular structures [92] and constitutes a useful asset in studies that require the generation of reporter lines, protein tagging, labelling of cellular structures, and lineage tracing experiments and shows promise for the advancement of cancer research [91].

The incorporation of inheritable cell-specific DNA barcodes in lineage tracing, followed by barcode sequencing, enables profiling of a large amount of of individual cells across various differentiation stages concurrently [93] and offers unprecedented insights into cellular dynamics and developmental processes [94].This technique provides a comprehensive picture of cellular dynamics within a tumor, allows to follow cell fate over extended periods, and different barcodes can be used to label and track distinct cell populations within the same sample, revealing interactions and relationships between them [94]. He et al. established a lineage recorder that combines reporter barcodes with inducible CRISPR–Cas9 scarring, which can be adapted in any iPSC-derived differentiation or organoid system to dissect lineage alterations during normal or perturbed development [95].

### Hypoxic PDAC organoids

The hypoxic TME of PDAC is known to cause EMT and resistance to therapy [70]. In most studies, PDAC organoids are cultured under normoxic conditions (O_2_ 20%), although intratumoral oxygen concentrations are estimated at O_2_ 0.7% [33, 38]. Geyer et al. cultured PDAC organoids under hypoxia (O_2_ 1%) and normoxia (O_2_ 20%), and analyzed their association with therapeutic resistance [35]. In order to understand the role of hypoxia related signaling in normoxic conditions, roxadustat (HIF-PHD inhibitor) was combined with gemcitabine [35]. In normoxia and hypoxia, roxadustat improved survival of mono- and cocultures. The responses to roxadustat propose that stabilization of HIF-1α in normoxia and in particular hypoxia contributes to limited response to gemcitabine, indicating a relevant role of PHD regulation and consequent HIF-1α signaling in PDAC [35]. They also postulate that hypoxia initiates specific molecular programs in PDAC organoids in mono- and cocultures (in a ratio of 2 (PDAC organoids): 1 (PSCs)) that effect response to different classes of compounds. These results proposed that targeting hypoxia driven signaling could lead to the effective targeting of tumor cells and potentially enhance response to conventional and targeted therapies [35]. Currently, there are no established protocols for isolating PDAC organoids from heterogeneous primary tumors under hypoxic conditions.

By subjecting organoids to selective cultivation under normoxic and hypoxic conditions, Kumano et al. successfully obtained two different clones regarding morphology, gene expression, and drug resistance [70]. Compared to normoxia-established pancreatic cancer organoids (NORMO-PCO), HYPO-PCO clones displayed distinct phenotypic shifts, including solid morphology and basal-like characteristics. Furthermore, these hypoxia-induced clones were characterized by higher expression of EMT-related genes and enhanced resistance to 5-FU, whereas NORMO-PCO clones remained cystic, classical, and sensitive to treatment. In addition, there are also differences in gene expression. While HYPO-PCO indicate KRAS-, TGF-β signaling, EMT, and Hedgehog, the NORMO-PCO had high expression of immune system-related genes, like IL-6, allograft, and coagulation [70]. These results indicate that NORMO-PCO and HYPO-PCO had matchless molecular signatures reflective of their respective oxygenation conditions [70]. As Moffitt et al. described, pancreatic cancer gene expression profiles can be stratified into two primary subtypes: the classical subtype, characterized by high expression of GATA binding protein 6 (GATA6) in NORMO-PCO tumors, and the basal-like subtype (HYPO-PCO), which is defined by low GATA6 expression [96, 97].

It should be highlighted that generation efficiency of organoids by establishment under normoxic conditions (O_2_ 20%) is not reflective of the hypoxic environment in which pancreatic cancer exists in patients [70]. To better imitate clinical conditions, the establishment of organoids under hypoxia in addition to normoxia and the investigation of hypoxia-resistant and drug-resistant cells that are responsible for the malignant phenotype of PDAC should be favored [70].

## Conclusions

PDAC is characterized by a high-density stroma, high interstitial pressure, and very low oxygen tension [35]. Based on better understanding of the regulatory mechanisms involved in primary and secondary resistance to systemic, immuno-, and radio-oncology approaches, it is necessary that both existing and novel therapeutic treatment options will be specified. Organoid models that replicate physiochemical characteristics, hypoxia, and stromal abnormalities of the TME are the most auspicious technology to provide information about the complexity of the tumorigenesis of PDAC, for promising therapy approaches and the establishment of an organoid biobank for precision medicine for PDAC. Schuth et al. demonstrate increased chemoresistance of PDAC organoids in coculture with patient-matched CAFs, emphasizing the relevance of complex coculture models for personalized medicine applications. This also opens the possibility to investigate efficacy and mode of action for drugs targeting the TME in a patient-specific way [25]. Since organoids are devoid of the native microenvironment comprising of stromal cells, muscle cells, blood vessels, and immune cells, the development of coculture conditions of organoids with immune cells or other cell types gain in importance [69]. PDO profiling using next generation sequencing of DNA and RNA combined with pharmacotyping may predict responses in pancreatic cancer patients and provide a rational for prioritizing therapeutic regimens [4]. In this way, the path to personalized medicine can be leveled. It has been already published, that the organoid technology is scheduled to forecast future clinical success through the results generated in vitro. However, it requires further validation, before functional testing can be applied in the clinic and it remains thrilling, whether PDOs can be used to guide therapy decisions [98]. In addition, both targeting hypoxic signaling and altering the TME to be less immunosuppressive and converting PDAC into an immunologically active tumor, hold potential for the treatment of PDAC. Prospective investigations should focus on targeting the entire tumor environment, including cancer cells, TME, immune system, and the hypoxia inducible modifications, responsible for the limited therapeutic response. The appropriate co-cultivation of several different cell types—such as CAFs and immune cells—that play a crucial role in the pathogenesis of pancreatic cancer remains a major challenge. Methodologically, conventional organoid culture should be expanded on synergistic combinations with advanced engineering-based technologies, advancing computational tools, developing novel barcode libraries, and integrating multiomics approaches and spatial transcriptomics. Organ-on-a-chip systems provide exceptionally controllable conditions with different human cell types and high reproducibility, and can also be combined with hypoxia to further enhance the validity of the results. FNA and CTCs can serve as readily available source material obtained directly from the patient and require only minimal cell quantities to enable a wide range of tests. The fusion of the two cutting-edge technologies, organoids and organ-on-a-chip systems, is also referred to as organoids-on-a-chip and is gaining prominence. At the same time, this combination solves numerous problems, such as nutrient supply and the simulation of realistic stimuli, while preserving the organoids’ patient-specific gene expression. In sum, PDAC organoid models offer unprecedented opportunities to dissect tumor biology and guide personalized therapy, but their translational utility hinges on resolving key challenges in mimicking the native TME. Integrated co-culture platforms can help simulate crosstalk between different cell types and organs while numerous powerful cutting-edge technologies demonstrate immense potential, and their strategic combination is expected to make a decisive contribution to finally closing the translational gap in pancreatic cancer.

## Data Availability

No datasets were generated or analysed during the current study.

## Abbreviations

ACC: Acinar cell carcinoma
ACP: Anaplastic carcinoma of the pancreas
ADM: Acinar-to-ductal metaplasia
ALK: Anaplastic lymphoma kinase
ALK5: Activin-like kinase 5
α-SMA: Alpha-smooth muscle actin
ApoA1: Apolipoprotein A1
ApoA2: Apolipoprotein A2
ARNT: Aryl hydrocarbon receptor nuclear translocator protein
ASC: Adenosquamous carcinoma
ASCs: Adult-derived stem/progenitor cells
BME: Basement membrane extract
BRCA1/2: Breast cancer susceptibility gene 1/2
BTK: Bruton tyrosine kinase
CA-125: Cancer-antigen 125
CA19-9: Carbohydrate antigen 19 − 9
CA242: Sialic acid-containing carbohydrate antigen 242
CAF: Cancer-associated fibroblast
CAR: Chimeric antigen receptor
Cas: CRISPR-associated protein
CDK1/2/4/6: Cyclin-dependent kinase 1/2/4/6
CDKN2A: Cyclin-dependent kinase inhibitor 2 A gene
CEA: Carcinoembryonic antigen
C-TAD: C-terminal transactivation domain
CREB: cAMP response element binding protein
CRISPR: Clustered regularly interspaced short palindromic repeats
CTGF: Connective tissue growth factor
CTL: Cytotoxic T-lymphocyte
CTNNB1: Catenin beta 1
DDR: DNA damage repair
ECM: Extracellular matrix
EGF: Epidermal growth factor
EGFR: Epidermal growth factor receptor
EMT: Epithelial-to-mesenchymal transition
ESC: Embryonic stem cell
FAK: Focal adhesion kinase
FAP: Fibroblast activation protein alpha
FBS: Fetale bovine serum
FDA: U.S. Food and Drug Administration
FGF2: Fibroblast growth factor 2
FIH: Factor-inhibiting hypoxia-inducible factor
FNA: Fine-needle aspiration
FOLFIRINOX: 5-Fluorouracil (5-FU), leucovorin, irinotecan, and oxaliplatin
GATA6: GATA binding protein 6
GNAS: Guanine nucleotide binding protein, alpha stimulating activity polypeptide
HAP: Hypoxia-activated prodrug
HBEGF: Heparin-binding epidermal growth factor (EGF)-like growth factor
HDACi: Histone deacetylase inhibitor
HER-2: Human epidermal growth factor receptor 2
HGF: Hepatocyte growth factor
HIF: Hypoxia-inducible factor
HRE: Hypoxia response elements
HRL: Hypoxia-responsive long non-coding RNA
HYPO-PCO: Pancreatic cancer organoids under hypoxia
ICI: Immune-checkpoint inhibitor
iPSC: Induced pluripotent stem cells
IPMN: Intraductal papillary mucinous neoplasia
ITGA4: Integrin alpha 4
ITPN: Intraductal tubular papillary neoplasm
KRAS: Kirsten rat sarcoma oncogene
LAMA5: Laminin alpha 5
LGALS9: Lectin galactoside-binding soluble 9
LGR5: Leucine-rich repeat containing G protein-coupled receptor 5
MCN: Pancreatic mucinous cystic neoplasm
MLSN: Melastatin
NAT: Neoadjuvant chemotherapy
NO: Nitric oxide
NORMO-PCO: Pancreatic cancer organoids under normoxia
NOS: Nitric oxide synthase
N-TAD: N-terminal transactivation domain
NTRK: Neurotrophic tyrosine receptor kinase
ODD: Oxygen-dependent degradation
p300/CBP: E1A binding protein p300/cAMP response element-binding protein
PALB2: Partner and localizer of BRCA2
PanIN: Pancreatic intraepithelial neoplasia
PARP: Poly(ADP-ribose) polymerase
PAS-A and PAS-B: Per-Arnt-Sim domains
PCO: Pancreatic cancer organoids
PDAC: Pancreatic ductal adenocarcinoma
PDO: Patient-derived organoid
PEG: Polyethylene glycol
PEGPH20: Pegvorhyaluronidase alfa
PFS: Progression free survival
PGE2: Prostaglandin E2
PHD: Prolyl hydroxylase domain
PSC: Pancreatic stellate cell
PSCA: Prostate stem cell antigen
pVHL-E3: Von Hippel-Lindau tumor suppressor protein-E3 ubiquitin ligase complex
Rex-1: Reduced expression protein 1
RNF43: Ring finger protein 43
ROCK: Rho-associated, coiled-coil-containing protein kinase
ROS: Reactive oxygen species
scRNA-seq: Single-cell RNA sequencing
SENP: Sentrin/SUMO-specific protease
SMAD4: Small mothers against decapentaplegic homolog 4 gene
SNO: S-nitrosylation
Sox-2: Sex determining region Y-box 2
STAT3: Signal transducer and activator of transcription 3
SUMOylation: Conjugation of small ubiquitin-related modifier protein
TGF-β1: Transforming growth factor beta 1
TIL: Tumor-infiltrating lymphocytes
TME: Ttumor microenvironment
TNF-α: Tumor necrosis factor alpha
TP53: Tumor protein 53 gene
Trop2: Trophoblast cell-surface antigen 2
TTR: Transthyretin
VEGF: Vascular endothelial growth factor
VEGF-A: Vascular endothelial growth factor A
Wnt: Wingless/integrase-1
